# cGAMP/Saponin Adjuvant Combination Improves Protective Response to Influenza Vaccination by Microneedle Patch in an Aged Mouse Model

**DOI:** 10.3389/fimmu.2020.583251

**Published:** 2021-02-02

**Authors:** Elena V. Vassilieva, Song Li, Heorhiy Korniychuk, Dahnide M. Taylor, Shelly Wang, Mark R. Prausnitz, Richard W. Compans

**Affiliations:** ^1^Department of Microbiology & Immunology and Emory Vaccine Center, Emory University School of Medicine, Atlanta, GA, United States; ^2^School of Chemical and Biomolecular Engineering, Georgia Institute of Technology, Atlanta, GA, United States

**Keywords:** microneedle vaccination, aged mice, Quil-A, cGAMP, combination adjuvant

## Abstract

Current strategies for improving protective response to influenza vaccines during immunosenescence do not adequately protect individuals over 65 years of age. Here, we used an aged mouse model to investigate the potential of co-delivery of influenza vaccine with the recently identified combination of a saponin adjuvant Quil-A and an activator of the STING pathway, 2’3 cyclic guanosine monophosphate–adenosine monophosphate (cGAMP) *via* dissolving microneedle patches (MNPs) applied to skin. We demonstrate that synergy between the two adjuvant components is observed after their incorporation with H1N1 vaccine into MNPs as revealed by analysis of the immune responses in adult mice. Aged 21-month-old mice were found to be completely protected against live influenza challenge after vaccination with the MNPs adjuvanted with the Quil-A/cGAMP combination (5 µg each) and demonstrated significantly reduced morbidity compared to the observed responses in these mice vaccinated with unadjuvanted MNPs. Analysis of the lung lysates of the surviving aged mice post challenge revealed the lowest level of residual inflammation in the adjuvanted groups. We conclude that combining influenza vaccine with a STING pathway activator and saponin-based adjuvant in MNPs is a novel option for skin vaccination of the immunosenescent population, which is at high risk for influenza.

## Introduction

The Global Influenza Mortality project estimated on average 389,000 influenza-associated respiratory deaths annually, of which 67% were among people 65 years and older (1). In the United States, the yearly vaccination rate of the population over 65 years old is estimated to be between 60% and 70%; however nearly 90% of influenza-related deaths occur in this age group (2). These numbers clearly demonstrate an inadequate protective immunity elicited by current influenza vaccines in immunosenescent individuals, and underscore the importance of research aimed at improving vaccines specifically designed for aged individuals. Recently, using an aged mouse model, we demonstrated that a novel combination adjuvant consisting of saponin Quil-A and a STING pathway activator cGAMP is more effective than the current methods in improving the reduced immune response in immunosenescence (3). In the present study we investigate the potential of delivering this adjuvanted formulation to the skin by means of dissolving microneedle patches (MNPs) (4).

Skin vaccination against influenza using MNPs (5, 6) has advanced to the stage of clinical trials (7–9). The MNPs used in this study are skin patches that contain an array of 100 solid, conical microneedles measuring hundreds of microns in length, and are made of water-soluble materials that encapsulate the vaccine and adjuvant(s). Upon application to skin, the microneedles painlessly penetrate the skin’s upper layers where they dissolve within minutes, thereby delivering the vaccine and candidate adjuvant(s). By targeting the skin, vaccination by MNPs has been shown to have a number of immunological advantages in comparison to traditional intramuscular administration in adult and young mouse models including dose sparing, stronger humoral and cellular immune responses, greater duration of immunity and broader cross-protection against heterologous virus strains (10–17). In addition, MNPs as a delivery system are generally preferred by patients (18–20) including older people (21). We have previously reported that MNP vaccination decreased the age—dependent decline of the functional antibodies compared to intramuscular injection. However, the “physical adjuvancy” effect produced by the mechanical placement of MNPs into the skin was diminished in the middle-aged and especially aged animals in which the antibody response was similar after MNP and IM vaccination (22). Thus, similar to systemic formulations MNPs need to be adjuvanted for successful use in the aged. Thermostability, an absence of sharps waste, as well as the possibility of self-administration represent some logistical advantages of MNPs. The adjuvanted MNPs are attractive as a way to avoid systemic reactogenicity often associated with the administration of adjuvants.

An agonist of the intracellular stimulator of interferon genes (STING) pathway, cGAMP, has been proposed as a skin adjuvant (23). Until recently it was unclear how efficiently charged dinucleotides such as cGAMP traverse the cell membrane to interact with intracellular STING, but a folate-organic phosphate antiporter SLC19A1 has now been identified as the major transporter of cyclic di-nucleotides in humans (24). We proposed that availability of charged compound cGAMP for an intracellular STING adaptor is increased by the addition of the membrane-active saponin to the vaccine formulation (3). In the present study, we co-incorporated cGAMP, the saponin adjuvant Quil-A, and a subunit influenza vaccine into dissolving MNPs, and assessed their immunogenicity and protective efficacy in live virus challenge experiments in the mouse model.

## Materials and Methods

### Ethics Statement

All institutional and national guidelines for the care and use of laboratory animals were followed in accordance with and approved by the Institutional Animal Care and Use Committees (IACUC) at Emory University and Georgia Institute of Technology.

### Animals

BALB/cAnNCrl female mice (Charles River Labs, Wilmington, MA) were housed in microisolators with filter tops in a biocontainment level BSL-1 animal facility and subjected to a 12/12-h light/dark cycle and temperature between 20 and 22^◦^C. Mice were moved to a BSL-2 facility operating under the same light and temperature conditions for challenge study. Young adult mice were 10 weeks old and aged mice were 21 months old by the time of immunization. Mice were randomly assigned to the groups and no animals were excluded from the study. Blinding was not possible due to small number of investigators.

### Viruses

H1N1 influenza A/California/07/09 virus obtained from the Centers for Disease Control and Prevention (CDC, Atlanta, GA), and H1N1 influenza A/Christchurch/16/10 virus NIB74 obtained from the National Institute for Biological Standards and Control (NIBSC, Potters Bar, UK) were expanded and titrated in MDCK cells (ATCC CCL 34 Manassas, VA) (22). Mouse-adapted A/California/07/09 H1N1 virus (25) was used in challenge experiments. The LD_50_ dose was determined in adult female BALB/c mice using the Reed-Muench calculation method (26).

### Vaccines

Influenza A (H1N1) 2009 monovalent A/California/07/09 H1N1 vaccine from BEI resources (NR-20347, Manassas, VA) and A/Christchurch/16/2010 NIB-74 (H1N1) vaccine monobulk generously provided by Seqirus (formerly NVS Influenza Vaccines, Cambridge, MA) were concentrated by ultrafiltration using Amicon Ultracel 30,000 MWCO spin filters and supplemented with 50 mM K-phosphate buffer, pH 7.4. A/Christchurch H1N1/16/10 was concentrated 11 x from 0.359 mg/ml HA to 4 mg/ml HA and A/California 07/09 H1N1 was concentrated 12–19 x from 0.03 mg/ml HA to 0.368 mg/ml HA or 0.574 mg/ml HA, respectively, and the stocks were combined and HA content was determined by SRID assay as previously described (27, 28) using strain-specific reagents from the Center for Biologics Evaluation and Research (Kensington, MD). These vaccines are pandemic-type 2009 strains protective against challenge with A/California/07/09 H1N1 virus.

### Adjuvants

Quil-A and 2’3’-cGAMP (cyclic [G(2’,5’)pA(3’,5’)p]), were purchased from InvivoGen (San Diego, CA). The stock solutions of adjuvants were prepared in 50 mM potassium phosphate buffer, pH 7.4.

### MNP Fabrication and Vaccine Delivery Efficiency

Dissolving MNPs were made in a two-step micromolding method as previously described (22). Adjuvanted or unadjuvanted MNPs had different formulations only in the first step. Briefly, a vaccine-loaded solution was casted on polydimethylsiloxane (PDMS) molds under vacuum. This casting solution consisted of vaccine (0.38 mg/ml A/Christchurch/16/10, or 0.29 mg/ml A/California/07/09), 1% w/v polyvinyl alcohol (PVA) and 10% w/v sucrose. When making adjuvanted MNPs, this solution contained additionally 0.48 mg/ml Quil-A and/or 0.34–3.4 mg/ml cGAMP for A/Christchurch MNPs, or 1.4 mg/ml Quil-A and/or 0.7–1.4 mg/ml cGAMP for A/California MNPs. The first step took 30 min under vacuum. In the second step, a polymer solution consisting of 18% w/w PVA and 18% w/w sucrose was cast on the mold to form the backing layer of MNPs. The filled molds were kept under vacuum for 3 h and on a 40°C hot plate overnight to completely dry the MNPs. Each MNP consisted of a 10 × 10 array of MNs within a square with around 7 mm sides (i.e., ~0.5 cm^2^). Each conical MN base diameter ≈ 200 µm, height ≈ 600 µm) was mounted atop an expanding pedestal (base diameter ≈ 600 µm, height ≈ 400 µm) (**Supplemental Figure 1**). All MNPs were stored with desiccant in individual sealed pouches until use. Vaccine content in the unadjuvanted MNPs was measured by ELISA using anti-HA A/California/07/09 antibodies (H1-Ab-1304 from the Center for Biologics Evaluation and Research (Kensington, MD) and SRID-measured vaccine stock as a standard, as previously described (22). When both saponin and cGAMP were present, the amount of the vaccine measured in MNPs was significantly lower than the unadjuvanted samples (**Supplemental Figure 2**) indicating that these adjuvants interfered with the quantification. Since all MNPs were prepared with the same volume of vaccines in one batch fabrication the amounts of Quil-A and cGAMP was calculated from the added stocks. Vaccine delivery efficiency determined by comparing vaccine content in unused vs. used unadjuvanted MNP was 70 ± 19% (mean ± SD, n=6) for A/Christchurch/16/2010-loaded MNPs used to vaccinate mature adult mice and 63 ± 12% for A/California07/09-loaded MNPs used to vaccinate the aged mice (**Supplemental Figure 1**).

### Vaccination Protocol

Mice were immunized once either intramuscularly (IM) in the upper quadrant of the hind leg or via MNPs applied to the depilated dorsal skin as described previously (27). The patches were applied to the skin of anesthetized mice using manual pressure for 1 minute and left on skin for total 20 minutes. The delivered vaccine dose was 1.3 ± 0.4 µg hemagglutinin (HA; mean, s.d., n=6) for A/ Christchurch/16/10 (H1N1) MNPs and 0.9 ± 0.2 µg HA (mean, s.d., n=11) for A/California/07/09 (H1N1) MNPs. IM vaccination with A/ Christchurch/16/10 (H1N1) vaccine was performed by intramuscular injection of 1.2 µg of vaccine antigen in a total volume of 0.05 ml.

### Challenge Study

Mice were challenged with mouse-adapted A/Ca07/09 (H1N1) virus (25) by intranasal installation of 30 µl of the virus stock under brief isoflurane anesthesia and monitored for the signs of infection as previously described (27) at 7 (young adult mice) or 6 (aged mice) weeks after single vaccination. The humane endpoint used for euthanasia was 25% loss of the initial body weight.

### Cytokine Assay in the Lungs

Lungs were collected from the aged mice that survived viral challenge and pushed through 40 µm cell strainers (VWR, Radnor, PA) in 1 ml of RPMI 1640 media (Mediatech, Manassas, VA). Lung lysates were clarified by centrifugation at 10,000 x g for 10 min and cytokines were measured using Bio-Plex Pro mouse cytokine group 1 23-plex panel (Bio-Rad Laboratories, Hercules, CA) according to the manufacturer’s instructions. Cytokine concentrations were normalized per total protein measured using bicinchoninic acid (BCA) assay with bovine serum albumin as the standard (Thermo, Massachusetts, USA).

### Quantification of Humoral Response

Blood samples were collected by submandibular bleeding and analyzed as described previously (22). Briefly, vaccine-specific antibody isotypes were determined by ELISA using Nunc MaxiSorp 96-well plates (ThermoFisher Scientific, Waltham, MA) coated with 100 ng HA of the same vaccine as used for immunization. Isotype standards and detection antibodies were from Southern Biotech, Birmingham, AL (capture goat anti-mouse antibodies #1010-01, isotype standards IgG # 010701, IgG1 #0102-01, IgG2a #0103-01, IgM #0101-01; HPR-conjugated goat anti-mouse secondary antibodies anti-IgG, #1030-05, anti-IgG1 #1070-05, anti-IgG2a #1080-05, anti-IgM #1020-05). Hemagglutination inhibition **(**HAI) titers were assessed based on the WHO protocol (29) using turkey red blood cells (Lampire biological laboratories, Pipersville, PA). The samples below the lowest level of detection (HAI = 10) were assigned a titer of 5 for calculations.

### Statistics

The statistical significance was calculated by one-way analysis of variance (ANOVA) with Tukey’s posttest using GraphPad Prism 8 software and *p* ≤ 0.05 was considered significant. HAI titers were converted to log2 for statistical analysis. We used software Statistica 7.0 (StatSoft, USA) to calculate the required sample size per group for α=0.05 and power goal 0.8 based on the parameters of immune response from our previous study (3). For adult mice we used means and SD of HAI titers at day 28 postvaccination for 1-way 7-group (1 non-adjuvanted and 6 adjuvanted) ANOVA analysis and for the aged mice we used means and SD of vaccine-specific IgG levels at day 28 postvaccination for 1-way 4-group (1 non-adjuvanted and 3 adjuvanted) ANOVA. The sample size of 4 for the adult mice and 7 for the aged mice was sufficient to observe significant differences in these parameters.

## Results

### Adjuvant Effect of cGAMP/Quil-A Combination Is Preserved in MNP-Vaccinated Young Adult 10-Week-Old Mice

**Figure 1:**
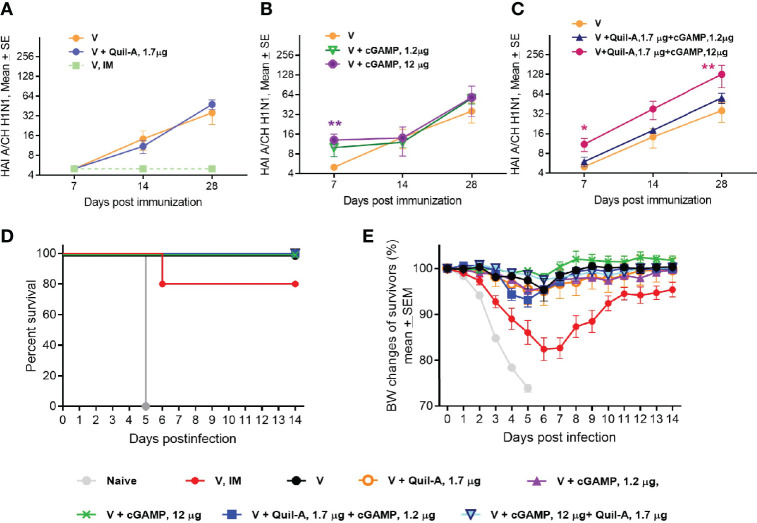
Effect of MNP vaccination in young adult 10-week-old mice (n=5 in all groups except the unadjuvanted MNP group, where n = 7). Mice were immunized once with A/Christchurch H1N1/16/10 (H1N1) vaccine alone or in combination with adjuvants by the IM route (1.2 µg HA, group V, IM) or through skin with MNP (1.3 ± 0.4 µg HA, all other groups). HAI titers were measured against A/Christchurch H1N1/16/10 (H1N1) virus. **(A)** HAI titers in mice immunized with unadjuvanted vaccine either IM or by MNPs or with MNPs which co-incorporated 1.2 µg of Quil-A with the vaccine; **(B)** HAI titers in mice MNP-vaccinated with either unadjuvanted vaccine or with vaccine combined with high (12 µg) or low (1.2 µg) doses of cGAMP; **(C)** HAI titers in mice MNP-vaccinated with either unadjuvanted vaccine or adjuvanted with a combination of cGAMP and Quil-A. The statistical significance between unadjuvanted and adjuvanted groups calculated by one-way ANOVA with Tukey’s posttest at the same time points postvaccination is represented by stars (*p < 0.05, **p < 0.01) **(D)** Survival of vaccinated mice challenged with a 47xLD_50_ dose of mouse-adapted A/California 07/09 H1N1virus. All mice were immunized with MNPs except for one group vaccinated with the unadjuvanted vaccine intramuscularly (V, IM). **(E)** Changes in body weight of vaccinated mice after challenge. Note that all unvaccinated mice succumbed to infection while all MNP-vaccinated mice survived. In the inadjuvanted IM group one mouse reached 25 % weight loss threshold at day 6 post challenge and was humanely euthanized.

We prepared MNPs by mixing concentrated A/Christchurch H1N1/16/10 (H1N1) vaccine with adjuvant stocks followed by micromold fabrication as described in the *Materials and Methods* section. Groups of 10-week-old BALB/c mice were vaccinated with MNPs that delivered 1.3 µg of an HA vaccine alone or in combination with low (1.2 µg) or high (12 µg) doses of cGAMP or with 1.7 µg Quil-A. A control group that received intramuscular (IM) injection of unadjuvanted vaccine (1.2 µg of HA) did not develop HAI titers, but most mice in the group that received unadjuvanted vaccine *via* MNPs developed HAI titers at or above the detection level of 10 by week 2 postvaccination which further increased to a geometric mean HAI titer (GMT HAI) of 24 by day 28 postvaccination (**Figure 1A** and **Supplemental Table 2**). Thus, in adult mice MNP delivery significantly improved antibody response to unadjuvanted vaccine, which was much greater than after vaccination by IM injection (GMT HAI 5 vs. 24, respectively, p = 0.0035 at day 28 postvaccination). Use of MNPs that contained vaccine and 1.7 µg of Quil-A as adjuvant did not change the dynamics of HAI titers compared to unadjuvanted MNPs (**Figure 1A**). cGAMP-loaded MNPs elicited HAI titers as soon as at day 7 postvaccination. However, in the absence of Quil-A, the low and the high doses of cGAMP elicited similar HAI titers (**Figure 1B**), potentially indicating a hindrance for the access of cGAMP to its intracellular receptor in the immunocompetent cells. Remarkably, the response to cGAMP was found to be dose-dependent upon addition of Quil-A. In the low dose cGAMP/Quil-A MNP group the titers did not differ from an unadjuvanted vaccine, but in the high dose cGAMP/Quil-A MNP group they were significantly higher than in the unadjuvanted MNP group at days 7 and 28 postvaccination (**Figure 1C**). A single application of MNPs containing 12 µg cGAMP + 1.7 µg Quil-A yielded the highest HAI GMT of 105.6, which was 4-fold higher by day 28 than that observed in the unadjuvanted MNP group (p = 0.008). The titers in Quil-A -supplemented high-dose cGAMP group demonstrated higher trend compared to the high-dose cGAMP only group (GMT HAI was 30.3 vs. 10, p = 0.057, at day 14 and 105.5 vs. 34.8, p = 0.14, at day 28 postvaccination, respectively). Thus the analysis of the dynamics of HAI titers revealed that in the adult mice, each adjuvant by itself induced lower HAI titers than in a combination. Levels of vaccine-specific immunoglobulins were the lowest in the systemically vaccinated mice and the highest in the combination MNP group. In the absence of Quil-A they were similar in the low and the high-dose cGAMP groups while addition of Quil-A significantly increased the level of total antibodies, especially IgG2a isotype (**Supplemental Figure 3**).

Mice were challenged with A/California07/09 H1N1 virus 1.5 months after single immunization. All non-vaccinated animals and one out of five mice vaccinated by the IM delivery route succumbed to infection (**Figure 1D**), while all groups of MNPs-vaccinated mice were completely protected. At day 6 postchallenge the animals in the IM group demonstrated the largest 18% drop in body weight and all MNP vaccinated animals exhibited a lower ~ 2%–5% weight drop (**Figure 1E**) confirming our previous findings of improved immunogenicity of MNP-delivered vaccine as compared to IM injection (25, 27). Altogether, the data obtained with young adult mice indicate that cGAMP and Quil-A were successfully incorporated into MNPs to generate strong immune responses to the HA antigen, which is consistent with previous works in which each adjuvant retained activity when used in MNP format (30, 31).

### Effect of Immunization of Aged Mice With Quil-A Alone-Adjuvanted or cGAMP/Quil-A-Adjuvanted Vaccine Using MNPs

Next, we tested whether cGAMP/Quil-A-adjuvanted MNPs will enhance protective immunity in aged mice. We immunized groups of 21-month-old mice with MNPs formulated with 0.9 µg A/California/07/09 (H1N1) vaccine alone or with 5 µg Quil-A as adjuvant alone, or in combination with 2.5 or 5 µg cGAMP and challenged them with 16 plaque forming units (pfu) of the homologous H1N1 virus 6 weeks after single immunization. We did not include a vaccine/cGAMP group because we previously demonstrated that cGAMP in 5 µg dose without addition of Quil-A did not improve survival of immunized aged mice compared to the unadjuvanted vaccine (3).

**Figure 2:**
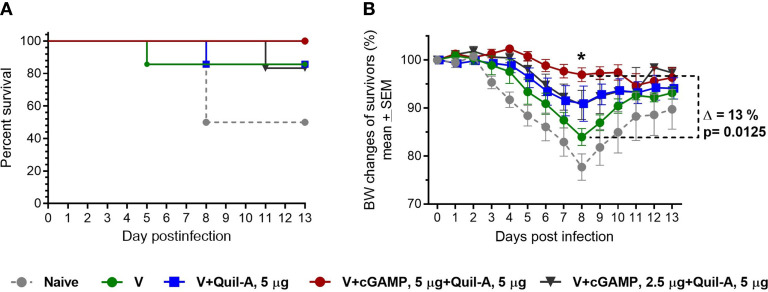
Effect of adjuvant composition of MNPs on protection of vaccinated aged mice against challenge. Aged 21-month-old animals received a single dose (0.9 ± 0.2 µg HA) of adjuvanted or non – adjuvanted A/California 07/09 H1N1 vaccine followed by challenge with 16 pfu of the mouse-adapted influenza A/California 07/09 H1N1virus 6 weeks postvaccination (n=7 in all groups except in the 5 µg Quil-A / 2.5 µg cGAMP group where one mouse expired, and in nonvaccinated group n = 6). **(A)** Survival, **(B)** Body weight of surviving mice. The statistical significance between unadjuvanted and adjuvanted MNP groups was calculated by one-way ANOVA with Tukey’s posttest (*p < 0.05 for Quil-A/cGAMP combination group (5 µg each).

**Figure 3:**
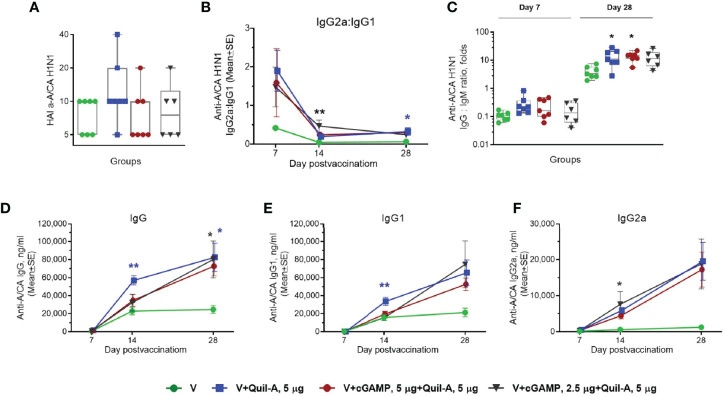
Effect of MNP immunization on the vaccine-specific humoral response in aged mice. Aged 21-month-old animals received a single dose (0.9 ± 0.2 µg HA) of adjuvanted or non–adjuvanted A/California 07/09 H1N1 vaccine. (n=7 in all groups except in the 5 µg Quil-A / 2.5 µg cGAMP group where 1 mouse expired n = 6). **(A)** Individual HAI titers at day 28 postvaccination. **(B)** Ratio of vaccine-specific IgG2a to IgG1. **(C)** Individual ratios of IgG to IgM on days 7 and 28 postvaccination; **(D)** Vaccine-specific IgG, **(E)** Vaccine-specific IgG1, **(F)** Vaccine-specific IgG2a. Stars indicate significance levels of the differences between adjuvanted and non-adjuvanted group at the same time postvaccination calculated by one-way ANOVA with Tukey’s posttest (*p < 0.05, **p < 0.01).

In the naïve group, three out of six (50%) mice survived the challenge. In the unadjuvanted group, six out of seven mice survived (**Figure 2A**) but exhibited a maximal average weight loss of 16% on day 8 postinfection (**Figure 2B**), indicating modest improvement in protective efficacy in the absence of adjuvant(s). Similar survival was observed in the groups that received Quil-A (5 µg) or Quil-A (5 µg)/cGAMP (2.5 µg)—adjuvanted MNPs, but the average weight loss was not significantly different from the unadjuvanted group (**Figures 2A, B**). Remarkably, the Quil-A/cGAMP combination group (5 µg each) was completely protected from mortality (**Figure 2A**) and demonstrated decreased morbidity as evidenced by only 3% maximal weight loss, that was 13% lower than in the non-adjuvanted group (p = 0.0125) (**Figures 2A, B**). Analysis of the immune responses to vaccination revealed that low HAI titers, characteristic of the aged mice (32, 33), did not correlate with protection (**Figure 2A** and **Supplemental Table 2**) or significantly differ between groups (**Figure 3A**). Compared to the nonadjuvanted MNPs, all adjuvanted MNPs demonstrated a trend of approximately 4-fold higher IgG2a/IgG1 ratio at day 7 postvaccination (**Figure 3B**), possibly indicating more efficient induction of a Th-1 based response in these groups. The ratio dropped from day 7 to days 14 and 28 but remained higher in the adjuvanted groups. It was 14 fold higher in Quil-A (5 µg)/cGAMP (2.5 µg) group by day 14 (p=0.003) and 5 fold higher in 5 µg Quil-A group by day 28 (p=0.033) compared to the non-adjuvanted MNP group. Quil-A (5 µg) and Quil-A /cGAMP (5 µg each) improved the isotype switch, as was indicated by ~3-fold higher IgG/IgM ratio by day 28 postvaccination (**Figure 3C**). The total levels of vaccine-specific IgG and its isotypes were higher in the adjuvanted groups vs. a non-adjuvanted group, with statistically significant differences in groups adjuvanted with 5 µg Quil-A alone and 5 µg Quil-A/2.5 µg cGAMP—combination (**Figures 3D–F**). Overall, the analysis of humoral immune responses revealed increased antibody production and class switch in all adjuvanted MNP groups compared to the non-adjuvanted group.

**Figure 4:**
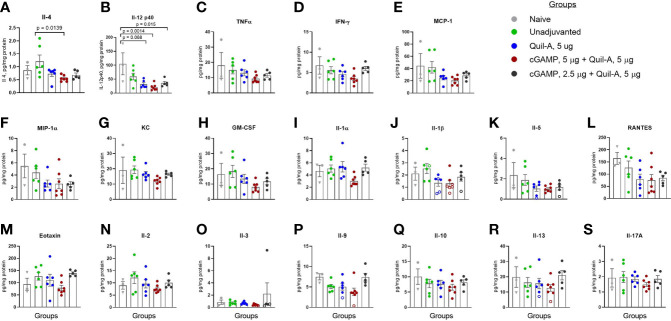
Effect of adjuvant composition of MNPs on the levels of cytokines and chemokines in lung lysates of the aged mice that survived the challenge. The lungs from all vaccinated mice and one naive mouse were collected on day 13 postchallenge (circles), and from the remaining two naïve mice on day 16 postchallenge (triangles). The open circles represent data extrapolated below standard range. **(A)** IL- 4; **(B)** IL-12 (p40); **(C)** TNF α; **(D)** IFN-γ; **(E)** MCP-1; **(F)** MIP-1α; **(G)** KC, **(H)** GM-CSF; **(I)** IL- 1α; **(J)** IL-1β; **(K)** IL- 5; **(L)** RANTES; **(M)** Eotaxin, **(N)** IL- 2, **(O)** IL- 3; **(P)** IL- 9; **(Q)** IL- 10, **(R)** IL- 13, **(S)** IL- 17A. Groups: Grey – naïve, n = 3; green – unadjuvanted vaccine MNP, n = 6; blue – vaccine adjuvanted with Quil-A, 5 μg MNP, n = 6; red – vaccine adjuvanted with cGAMP, 5 μg + Quil-A, 5 μg MNP, n = 6; black – vaccine adjuvanted with cGAMP, 2.5 μg + Quil-A, 5 μg MNP, n = 6. The statistical significance between groups was calculated by one-way ANOVA with Tukey’s posttest.

The surviving mice were sacrificed 2 weeks after challenge, and the lung lysates were analyzed for differences in the levels of cytokines and chemokines. Significant differences were observed in the levels of two interleukins: Il-4 was 2.2-fold lower in the 5 µg Quil-A/5 µg cGAMP combination group than in the unadjuvanted group (p = 0.0139) (**Figure 4A**), and compared to the naïve group Il-12 (p40) was 5-fold lower in 5/5 combination group (p = 0.0014) and ~3-fold lower in the Quil-A only (p = 0.0068) and 2.5/5 combination (p = 0.015) groups, with no significant differences between survivors in unadjuvanted and naïve groups (**Figure 4B**). The absence of significant changes in other tested cytokines (**Figure 4**) are most probably due to two factors: first, the lungs were analyzed at the resolving phase of infection in the surviving mice and second, the small number of survived naive animals, since 50% of them succumbed to infection. Nevertheless, there was a clear trend of the lowest levels of THFα, IFN-γ, MCP-1, MIP-1α, and KC in the cGAMP/Quil-A group, 5 ug each (red circles in **Figure 4**), in comparison to other MNP groups and the non-vaccinated group. We expected to observe markers of inflammation at the completion of challenge because it was previously reported that they lingered for longer time in the lungs of influenza-infected aged C57BL/6 mice compared to the young ones in which some inflammatory cytokines peaked at 6 days post-infection and then returned to baseline by day 12 as the infection was resolving but remained significantly higher on days 9 and 12 in aged 19–22-month-old mice (34). The reduced levels of these Th2 and Th1 cytokines in the lungs of recovered animals at the resolution of infection was consistent with the reduced morbidity estimated from the decrease in weight loss achieved with the adjuvanted MNPs, especially in the 5 µg Quil-A/5 µg cGAMP group. In summary, compared to the unadjuvanted MNP formulation, MNP delivery of the Quil-A/cGAMP (5 µg each)—adjuvanted vaccine not only completely protected aged mice from a physiologically relevant ~1xLD_50_ challenge dose but also reduced inflammation in the lungs postchallenge.

## Discussion

The aim of this study was to investigate the possibility of using cGAMP/Quil-A as a combination adjuvant with influenza vaccine in MNP format to enhance immune responses in aged mice. MNPs possess the unique property of “physical adjuvancy” which arises from the physical puncture of skin by microneedles during application of MNPs, which in turn induces limited (and painless) cell damage leading to inflammation at the site of application (35). Physical adjuvantation may also come from formulation of the MNPs using a water-soluble polymer that forms a gel upon contact with interstitial fluid in the skin. As the gel dissolves and the antigen and adjuvant(s) slowly diffuse away from the site of MNP application, the antigen and adjuvant(s) have a more extended presentation to the immune system, which can lead to improved immune responses (36). Other factors, such as the presence of skin-resident antigen-presenting cells, for example Langerhans cells and dermal dendritic cells, as well as lymphatics draining from the skin may also contribute to the efficacy of skin vaccination by MNPs (37, 38). As we confirmed in this work, simply changing the influenza vaccine delivery route from systemic vaccination to skin vaccination by MNPs increased vaccine efficacy in young adult mice (27). Experiments with the 10-week-old mice were performed to determine whether the adjuvant properties were preserved in MNPs. A direct comparison between adult and aged mice vaccinated with cGAMP/Quil-A combination was described previously (3) but it was not the goal of this current work. We did not include IM group in the aged mice study because we previously compared IM and MNP delivery methods and demonstrated that MNPs did not significantly improve vaccine immunogenicity in mice over 14 months old (22). Here we show that MNPs can be successfully used to co-incorporate a cGAMP/Quil-A adjuvant combination with influenza vaccine. This finding is consistent with published studies in which each adjuvant retained activity when used alone in MNP format (30, 31). We previously demonstrated that availability of cGAMP for an intracellular STING adaptor is increased by the addition of the membrane-active saponin to the vaccine formulation (3). The delivery of STING ligands inside cells can also be achieved using particle- and liposome-based delivery systems (39) many of which require special preparation procedures and are not compatible with microneedle format. A practical advantage of cGAMP/Quil-A combination is that it can be easily added to both liquid formulations and incorporated in the MNPs.

Mice between 18–24 month of age roughly correspond to 56–69-year-old humans age-wise (40). In our previous study the animals were 19-month-old at vaccination and 20-month-old by the time of challenge and in the current study they were 21-month-old at vaccination and 22.5-month-old by challenge. Because of more advanced age the immune response postvaccination was expectedly lower in these animals, but they were completely protected from a low dose challenge after a single vaccination with MNPs adjuvanted with the same Quil-A/cGAMP combination (5 μg each). The amount of HA antigen in our experiments was chosen on the basis of our previous data. Previously, 1 μg of non-adjuvanted H1N1 vaccine was not protective against high challenge dose (300 pfu), yet the addition of the combination adjuvant increased protection to 100% (3). Here, we used a lower challenge dose of 16 pfu at which 86% of vaccinated aged mice survived but exhibited high weight loss which was prevented by using cGAMP/Quil-A—adjuvanted MNPs for skin vaccination. Use of low infection dose ~ 1xLD_50_ in this study better resembled the outcome of flu infection in humans and allowed us to analyze the residual inflammation in the lungs of surviving mice in unadjuvanted and adjuvanted groups. The low level of Th1 and Th2 cytokines IL-12 and Il-4 at the resolving phase of infection in the surviving mice vaccinated with adjuvant combination indicated a lower degree of inflammation, consistent with the lowest weight loss in this group at the peak of infection. The HAI titers detected in all groups of aged mice were low consistent with published data (32, 33) and did not correlate with protection. The total vaccine-specific antibodies were generally higher in most adjuvanted groups. The cGAMP/Quil-A group, 5 µg each, that demonstrated complete survival and the lowest weight loss after challenge also presented with reduced inflammation markers as compared to other MNP groups. In elderly humans, T-cell responses better correlate with protection than antibody titers (41). Similarly, our data indicate possible involvement of T-cell–mediated immunity (34) stimulated by cGAMP/Quil-A combination that we plan to address as a future direction of research. The main goal of this study was to determine if the previously identified combination of cGAMP and Quil-A, 5 μg each (vaccine/adjuvant ratio ~1:5:5, wt/wt) will remain effective in MNPs. We previously determined that a 5 μg dose of cGAMP did not improve the protective immunity in the aged mice but was protective when combined with Quil-A (3). Quil-A is a mixture of saponins and as such has inherent safety concerns. In the preliminary screening, we observed a small scab on the skin surface at the site of intradermal injections of vaccine formulated with 10 μg Quil-A. Thus, we kept the amount of Quil-A delivered with MNPs to 5 μg which is similar to 3 μg dose recently used to adjuvant tetravalent Demge sE in Nanopatch (42). The visual inspection of the MNP application sites did not reveal skin irritation or scabs. QS-21 is a fractions Quil-A with lower toxicity (43) and potent adjuvancy (44). Nanopatch adjuvanted with 1.5 μg dose of OS-21 was effective in adult mice; increasing the dose up to 6 ug led to decrease of flu-specific antibodies (45). Further dose response studies involving QS-21 and other saponin compounds (46–49) as well as new and potent STING activators will define the optimal ratio between the active pharmaceutical ingredients to best balance between adjuvant activity and possible reactogenicity. Another important question to be answered in future research is breadth of immunity in aged animals vaccinated with adjuvanted MNPs by using heterologous challenge strains.

It is important for the skin vaccination format that human keratinocytes respond to cGAMP treatment (50). STING pathway activators are also effective as mucosal adjuvants, but mucosal delivery may potentially promote allergic asthma as was recently demonstrated in mice (51). Delivering STING agonists through the skin *via* MNPs may be a better approach: metal MNPs coated with such agonists were shown to elicit lower levels of antigen-specific IgE than when coated with Alum adjuvant (30). In a previous report (3) we demonstrated a combined effect of cGAMP and saponin in the aged but not in young mice. Although there is no clear explanation of this effect, a recent study demonstrated that tumor immune checkpoint blockade therapy was not effective in the aged mice without intratumoral stimulation of STING, but in younger mice the therapy itself was effective and STING activation did not increase the efficiency further (52).

The adjuvanted MNPs used in this study can be considered to represent a skin vaccine delivery system that combines physical adjuvantation with two chemical adjuvants. Given the immunological and logistical advantages of MNPs, they hold great promise as a modern vaccine delivery platform (6, 53, 54). For example, dissolving microneedles were employed to deliver a novel vaccine against COVID-19 coronavirus (55). Older people are receptive to using MNPs as a delivery technology (21) and our findings open a new vaccination option specific for the aged population at high risk for influenza. To our knowledge this is the first report that demonstrates the successful incorporation of a novel and effective cGAMP/Quil-A combination adjuvant into dissolving microneedle patches. This formulation improves protective efficacy of an influenza vaccine in aged mice. This is a significant result which indicates that the vaccination outcome in the elderly can be improved by the immune activating effects of this combination of adjuvants delivered together with licensed HA-based influenza vaccine *via* skin microneedle patches using a single vaccination approach which is consistent with the seasonal flu vaccination recommendations.

## Data Availability Statement

The original contributions presented in the study are included in the article/**Supplementary Material**. Further inquiries can be directed to the corresponding author.

## Ethics Statement

The animal study was reviewed and approved by the Institutional Animal Care and Use Committee (IACUC) at Emory University and Georgia Institute of Technology.

## Author Contributions

The experimental design was conceived by EV, RC, and MP. SL manufactured and analyzed MNPs. EV, DT, HK, and SW carried out animal experiments and analyzed the immune responses. The manuscript was written by EV, RC, and MP. All authors contributed to the article and approved the submitted version.

## Funding

This project has been funded by the National Institute of Allergy and Infectious Diseases, a component of the NIH, Department of Health and Human Services, under contract 75N93019C00052 and project number 5R01AI110680-05.

## Conflict of Interest

MP is an inventor of patents licensed to companies developing microneedle-based products, is a paid advisor to companies developing microneedle-based products and is a founder/shareholder of companies developing microneedle-based products (Micron Biomedical). This potential conflict of interest has been disclosed and is managed by Georgia Tech. RC and EV have filed a patent application for the adjuvant combination.

The remaining authors declare that the research was conducted in the absence of any commercial or financial relationships that could be construed as a potential conflict of interest.
