# Supplementary material for: cGAMP/Saponin Adjuvant Combination Improves Protective Response to Influenza Vaccination by Microneedle Patch in an Aged Mouse Model
Source: Front Immunol. 2021 Feb 2;11:583251. doi: 10.3389/fimmu.2020.583251 (PMC7884748; doi:10.3389/fimmu.2020.583251)
Supplement: Supplementary file 1 [file DataSheet_1.pdf]

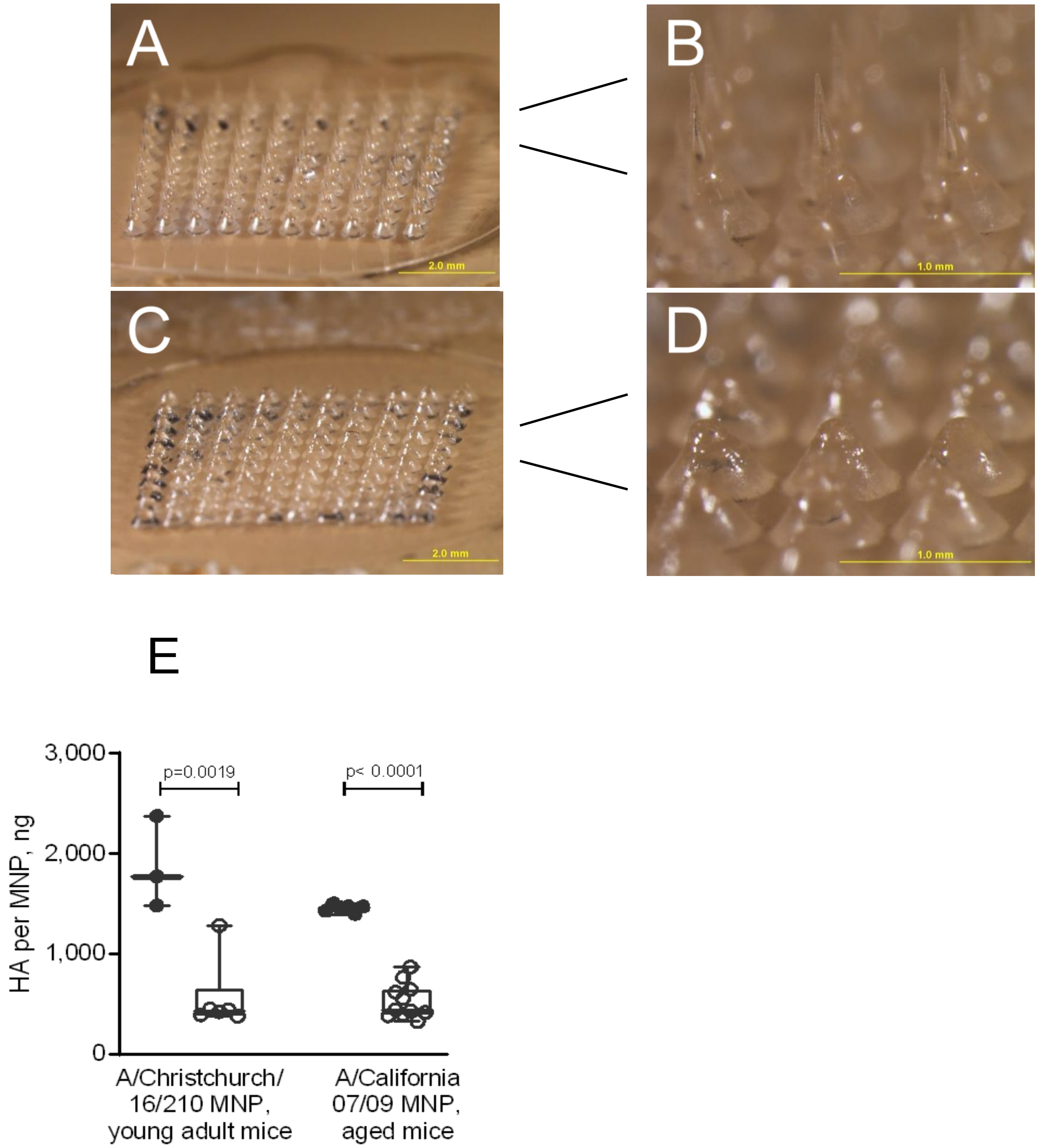

Supplementary Figure 1. Dissolving MNPs. A-D) Unused (A,B) and used (C,D) MNPs. Magnification 16 x (A,C) and 63 x (B,D). E). Vaccine delivery efficiency by MNPs. Vaccines were extracted from unadjuvanted MNPs before (filled circles) or after (open circles) they were used to vaccinate mice. HA content was measured by ELISA as previously described (1). In A/Christchurch/16/2010 H1N1 vaccine - loaded MNPs HA content was found to be 1.87 ± 0.45 µg per MNP before use (n=3), and 0.56 ± 0.35 µg per MNP after application to the skin of young adult mice (n=6). This corresponds to delivery of 1.3 ± 0.4 µg HA per MNP and a delivery efficiency 70 ± 19% (mean, SD, n=6). HA content in A/California07/09 H1N1 vaccine -loaded MNPs was found to be 1.45 ± 0.04 µg per MNP before use (n=6), and 0.53 ± 0.17 µg per MNP after application to the skin of the aged mice (n=11). This corresponds to delivery of 0.9 ± 0.2 µg HA per MNP and a delivery efficiency 63 ± 12% (mean, SD, n=6).

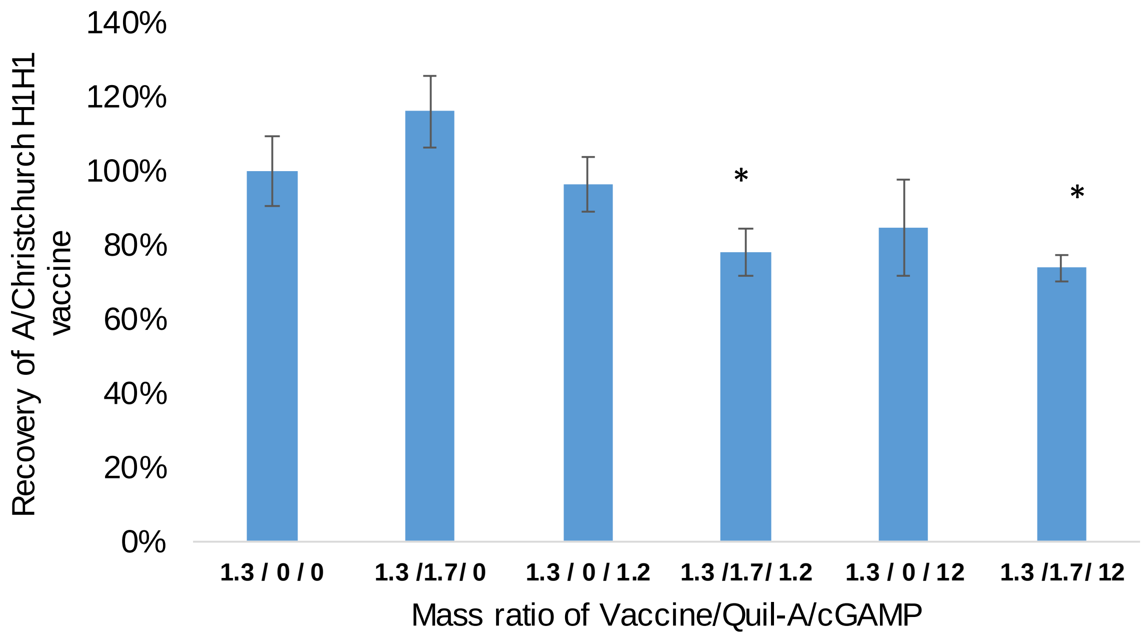

Supplementary Figure 2. Interference of adjuvants with vaccine quantification by ELISA in extracts of MNPs (n = 3 per group).

Supplemental Table 1. HAI titers at day 28 postvaccination in young adult mice

Formulation	Route	GMT*	95% CI
1.2 µg A/Christchurch/16/2010 H1N1	IM	5	*[5 - 5]*
1.3 µg A/Christchurch/16/2010 H1N1	MNP	24.4	*[10.1 – 59.1]*
1.3 µg A/Christchurch/16/2010 H1N1+ 1.7 µg Quil-A	MNP	46	*[31.3 – 67.5]*
1.3 µg A/Christchurch/16/2010 H1N1+ 1.2 µg cGAMP	MNP	52.8	*[32.9 – 84.6]*
1.3 µg A/Christchurch/16/2010 H1N1+ 12 µg cGAMP	MNP	35	*[8.5 – 143.2]*
1.3 µg A/Christchurch/16/2010 H1N1+ 1.2 µg cGAMP + 1.7 µg Quil-A	MNP	52.8	*[32.9 – 84.6]*
1.3 µg A/Christchurch/16/2010 H1N1+ 12 µg cGAMP + 1.7 µg Quil-A	MNP	105.6	*[48.9 – 227.9]*

* The samples below the lowest level of detection (HAI = 10) were assigned a titer of 5 for calculations

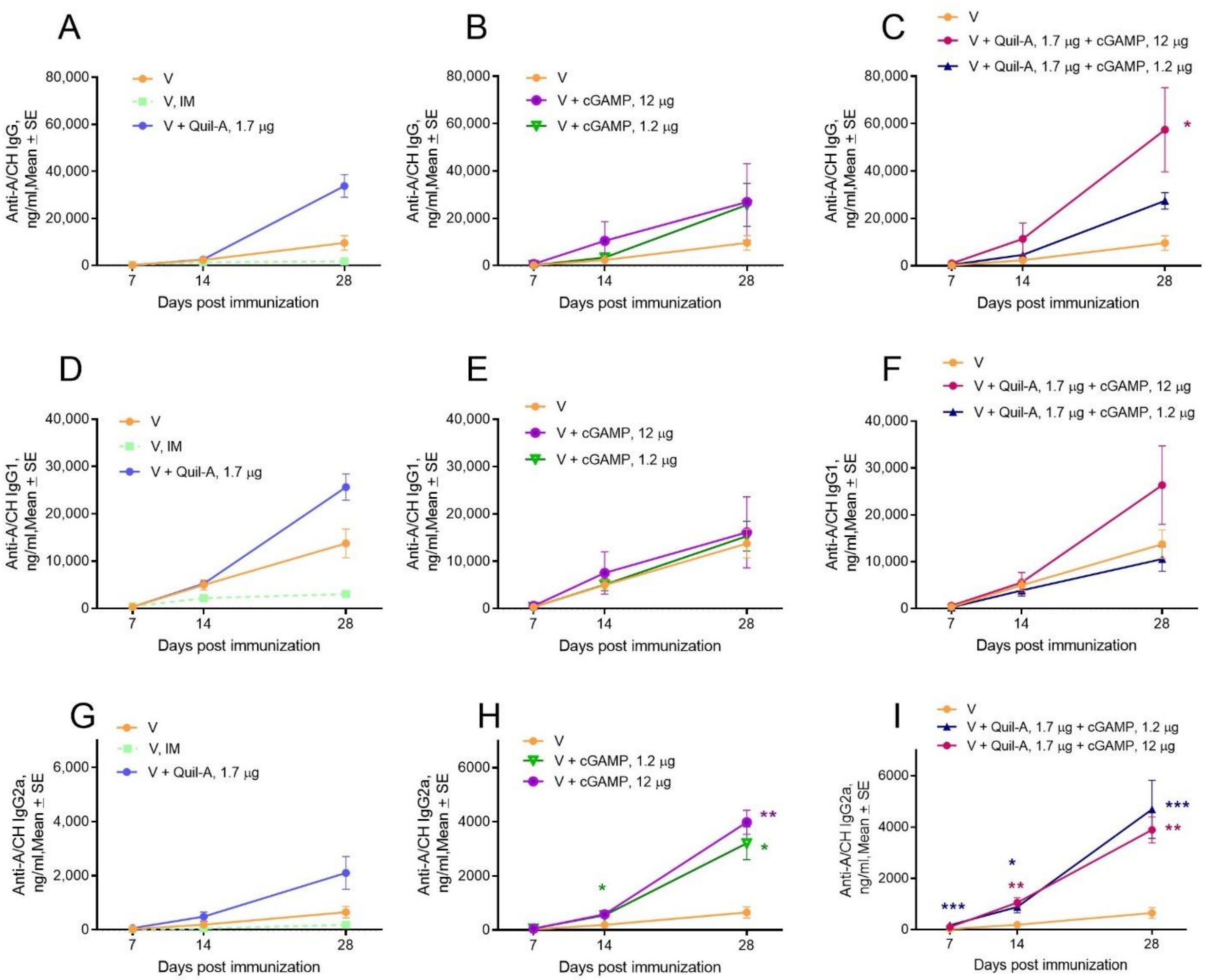

Supplemental Figure 3. Effect of MNP vaccination on vaccine-specific total antibody levels in young adult 10-week –old mice (n=5 in all groups except the unadjuvanted MNP group, where n = 7). Mice were immunized once with A/Christchurch H1N1/16/10 (H1N1) vaccine alone or in combination with adjuvants by the IM route (1.2 µg HA, group V, IM) or through skin with MNP (1.3 ± 0.4 µg HA, all other groups). Levels of vaccine – specific antibodies were detected by ELISA. A-C) IgG; D-F) IgG1, G-I) IgG2a. A,D,G) Mice immunized with unadjuvanted vaccine either IM or by MNPs or with MNPs which co-incorporated 1.2 µg of Quil-A with the vaccine; B,E,H) Mice MNP-vaccinated with either unadjuvanted vaccine or with vaccine combined with high (12 µg) or low (1.2 µg) doses of cGAMP; C,F,I) Mice MNP-vaccinated with either unadjuvanted vaccine or adjuvanted with a combination of cGAMP and Quil-A. The statistical significance between unadjuvanted and adjuvanted groups calculated by one-way ANOVA with Tukey's posttest at the same time points postvaccination is represented by stars (*p<0.05, **p<0.01).

Supplemental Table 2. HAI titers at day 28 postvaccination in aged mice

Formulation	Route	GMT*	95% CI
0.9 µg A/California07/09 H1N1	MNP	7.4	*[5.3 – 10.5]*
0.9 µg A/California07/09 H1N1+ 5 µg Quil-A	MNP	12.2	*[6.6 – 22.4]*
0.9 µg A/California07/09 H1N1+ 5 µg Quil-A + 5 µg cGAMP	MNP	7.4	*[4.5 – 12.3]*
0.9 µg A/California07/09 H1N1+ 5 µg Quil-A + 2.5 µg cGAMP	MNP	7.9	*[4.3 – 13.4]*

* The samples below the lowest level of detection (HAI = 10) were assigned a titer of 5 for calculations

Reference

1. E. V. Vassilieva, S. Wang, S. Li, M. R. Prausnitz and R. W. Compans: Skin immunization by microneedle patch overcomes statin-induced suppression of immune responses to influenza vaccine. *Sci Rep* (2017) 7(1): 17855 doi:10.1038/s41598-017-18140-0
